# Autophagy and LRRK2 in the Aging Brain

**DOI:** 10.3389/fnins.2019.01352

**Published:** 2019-12-17

**Authors:** Federica Albanese, Salvatore Novello, Michele Morari

**Affiliations:** ^1^Section of Pharmacology, Department of Medical Sciences, University of Ferrara, Ferrara, Italy; ^2^Laboratory of Molecular and Chemical Biology of Neurodegeneration, Brain Mind Institute, School of Life Sciences, École Polytechnique Fédérale de Lausanne, Lausanne, Switzerland

**Keywords:** aging, α-synuclein, autophagy, LC3, LRRK2, lysosomes, Parkinson’s disease, LAMP2A

## Abstract

Autophagy is a highly conserved process by which long-lived macromolecules, protein aggregates and dysfunctional/damaged organelles are delivered to lysosomes for degradation. Autophagy plays a crucial role in regulating protein quality control and cell homeostasis in response to energetic needs and environmental challenges. Indeed, activation of autophagy increases the life-span of living organisms, and impairment of autophagy is associated with several human disorders, among which neurodegenerative disorders of aging, such as Parkinson’s disease. These disorders are characterized by the accumulation of aggregates of aberrant or misfolded proteins that are toxic for neurons. Since aging is associated with impaired autophagy, autophagy inducers have been viewed as a strategy to counteract the age-related physiological decline in brain functions and emergence of neurodegenerative disorders. Parkinson’s disease is a hypokinetic, multisystemic disorder characterized by age-related, progressive degeneration of central and peripheral neuronal populations, associated with intraneuronal accumulation of proteinaceous aggregates mainly composed by the presynaptic protein α-synuclein. α-synuclein is a substrate of macroautophagy and chaperone-mediated autophagy (two major forms of autophagy), thus impairment of its clearance might favor the process of α-synuclein seeding and spreading that trigger and sustain the progression of this disorder. Genetic factors causing Parkinson’s disease have been identified, among which mutations in the LRRK2 gene, which encodes for a multidomain protein encompassing central GTPase and kinase domains, surrounded by protein-protein interaction domains. Six LRRK2 mutations have been pathogenically linked to Parkinson’s disease, the most frequent being the G2019S in the kinase domain. LRRK2-associated Parkinson’s disease is clinically and neuropathologically similar to idiopathic Parkinson’s disease, also showing age-dependency and incomplete penetrance. Several mechanisms have been proposed through which LRRK2 mutations can lead to Parkinson’s disease. The present article will focus on the evidence that LRRK2 and its mutants are associated with autophagy dysregulation. Studies in cell lines and neurons *in vitro* and in LRRK2 knock-out, knock-in, kinase-dead and transgenic animals *in vivo* will be reviewed. The role of aging in LRRK2-induced synucleinopathy will be discussed. Possible mechanisms underlying the LRRK2-mediated control over autophagy will be analyzed, and the contribution of autophagy dysregulation to the neurotoxic actions of LRRK2 will be examined.

## Introduction

Autophagy is a highly conserved cellular degradation process, either bulk or selective, by which long-lived macromolecules, protein aggregates and dysfunctional/damaged organelles are delivered to lysosomes for degradation (Deter et al., 1967; Mortimore and Schworer, 1977). This constitutive physiological activity works in parallel with the UPS to implement protein quality control and maintain the integrity of cell proteome (i.e., cellular proteostasis). Recently, the involvement of autophagy in general energy homeostasis has also been proven, since autophagy can be stimulated in response to starvation-induced stress, amino acid depletion as well as cell energy needs (Puente et al., 2016; Ho et al., 2017; Olsvik et al., 2019; Sutton et al., 2019).

Dysfunctions of the ALP have been reported in aging as well as in several human disorders, including cancer, chronic inflammatory diseases, cardiomyopathies and neurodegenerative diseases (Mizushima et al., 2008; Boland et al., 2018; Deretic and Klionsky, 2018; Onorati et al., 2018; Zech et al., 2019). Notably, neurodegenerative disorders of aging such as Parkinson’s disease (PD), Alzheimer’s disease (AD), Huntington’s disease (HD) frontotemporal dementia and amyotrophic lateral sclerosis are characterized by a common feature: accumulation of aberrant or misfolded proteins, such as α-synuclein (α-syn), Aβ, tau, mutant forms of huntingtin, TDP43, which are neurotoxic (Gal et al., 2009; Bourdenx et al., 2017; Boland et al., 2018). Furthermore, either blocking or promoting autophagy has an impact on clearance of cytotoxic proteins in different *in vitro* and *in vivo* models of aggregopathies/proteinopathies (Sarkar et al., 2008; Crews et al., 2010; Spilman et al., 2010; Ciechanover and Kwon, 2017; Moors et al., 2017).

## The Authophagy-Lysosomal Pathway

**FIGURE 1:**
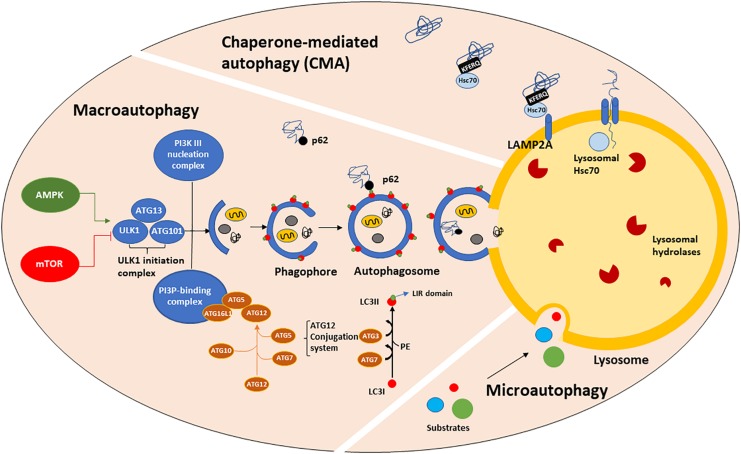
The autophagic machinery. Three types of autophagy have been described: macroautophagy (henceforth referred to as autophagy), chaperone-mediated autophagy (CMA) and microautophagy. Autophagy is positively regulated by AMPK and negatively by mTORC1 which, in turn phosphorylates and mediates ULK1-initiation complex association and activation. The ATGs protein family, organized in three major complexes (the ULK1-initiation complex, PI3K III nucleation complex and PI3P-binding complex), mediates the formation of a cup-shaped membrane, termed phagophore. The phagophore directly engulfs a small portion of cytoplasm containing damaged organelles, misfolded or old proteins to form the autophagosome. Then, the autophagosome fuses with the lysosome, generating the autolysosome, in which autophagic substrates are degraded by lysosomal hydrolases activity and acidic environment. Substrate recognition and sequestration can also occur in a selective manner through intervention of LC3II. LC3I is a cytosolic protein, which after being conjugated to PE, translocates onto the phagophore membranes where it acts as a receptor for multiple cargo proteins, such as p62. p62 binds both LC3II (LIR domain) and ubiquitinated proteins (UBA domain), allowing their entry into the autophagosome for degradation. CMA is a one-by-one fashion type of autophagy and it is only referred to proteins bearing the KFERQ-like motif. Those proteins are directly recognized by cytosolic Hsc70, which shuttles them to the lysosomal membrane where it binds LAMP2A, a transmembrane receptor. The binding of Hsc70 to LAMP2A leads to its oligomerization, promoting the entry of CMA substrates into the lysosomal lumen. The presence of lysosomal Hsc70 is also required for cargo sequestration. Microautophagy is the least characterized form of autophagy. During microautophagy, substrates are directly engulfed by the lysosomal membrane, without an intermediate autophagosome formation.

Macroautophagy, CMA and microautophagy are the three major forms of autophagy identified so far, although other forms such as selective and precision autophagy have been described more recently (Klionsky, 2005; Massey et al., 2006; Kimura et al., 2015; Dikic, 2017). Macroautophagy (henceforth referred to as autophagy) requires autophagosome biogenesis, a complex multi-step process regulated by the autophagy-related (ATG) gene family member proteins (Tsukada and Ohsumi, 1993; Thumm et al., 1994; Klionsky et al., 2003; Klionsky, 2012), whose transcription is driven by TFEB (Settembre et al., 2011) and many other transcription factors, such as FOXOs, E2F1, CREB, PPARγ to name a few (Fullgrabe et al., 2016). ATGs protein activity is controlled upstream by nutrient and growth signaling pathways. Autophagy starts with the formation of an isolation cup-shaped membrane (also termed phagophore) that elongates and sequesters a small portion of the cytoplasm to form the autophagosome (Figure 1). Then, the autophagosome fuses to the lysosomes, generating autolysosomes. Selective cargo recognition and sequestration into the autophagosome lumen require the presence of receptor-proteins, among which microtubule-associated protein 1 light chain 3 (known as MAP1LC3 or LC3). The cytosolic form of LC3, LC3I, translocates to the autophagosome membranes after being conjugated to phosphatidylethanolamine (Stolz et al., 2014; Rockenfeller et al., 2015). The abundance of LC3II, i.e., the lipidated form of LC3I, is directly correlated with the number of mature autophagosomes. LC3II partners in cargo recognition and delivery to lysosomes are a number of selective-autophagy receptor proteins such as Sequestosome 1 (SQSTM1)/p62 and other sequestosome 1-like receptors (NBR1, optineurin, NDP52, TAX1BP1, TOLLIP and ALFY/WDFY3) (Conway et al., 2019). p62, the first autophagy adaptor protein to be identified (Ishii et al., 1996), recognizes ubiquitinated proteins via its ubiquitin-associated (UBA) domain and docks onto the forming phagophore membrane through binding LC3II via the LC3-interacting region (LIR). Impaired autophagy leads to SQSTM1/p62 accumulation and aggregation of ubiquitinated proteins (Komatsu et al., 2007a). LC3I, LC3II, SQSTM1/p62 and mTOR are all validated markers of autophagy (Brown et al., 1994; Sugawara et al., 2004; Lippai and Low, 2014).

Mammalian target of rapamycin complex 1 (mTORC1) and 5′-AMP-activated kinase (AMPK) act as key upstream regulators of autophagy by modulating the serine-threonine kinase activity of ULK1/2. Upon activation, ULK1 phosphorylates and associates with ATG101, focal adhesion kinase family interacting protein of 200 kDa (FIP200) and ATG13 (Hara et al., 2008; Chan, 2009; Ganley et al., 2009) to generate the Initiation Complex. This complex acts as cellular sensor for glucose, nitrogen, growth factors, amino acids and ROS concentrations (Kim and Guan, 2015). In the presence of high levels of nutrients, mTORC1 acts as an inhibitor of autophagy by directly hyperphosphorylating ATG13 and ULK1 (at Ser757), which prevents ULK1 binding to ATG13 and AMPK (Kim and Guan, 2015; Meijer et al., 2015). Conversely, amino acids depletion suppresses mTORC1 activity. Furthermore, there is evidence that under glucose starvation or increased AMP/ATP ratio, AMPK negatively modulates mTORC1 pathway, phosphorylating (at Ser317 and Ser777) and activating ULK1 (Kim et al., 2011). Interestingly, recent studies have shown that amino acid and glucose depletion promote autophagy with different efficiencies. Indeed, amino acid starvation is able to induce ULK1-initiation complex formation and promote a great amount of autophagosomes containing LC3II and p62 whereas glucose starvation does not (Nwadike et al., 2018). In addition, amino acid but not glucose depletion, leads to lysosomal acidification, independently from autophagy and ULK1 (Nwadike et al., 2018). Furthermore, there is evidence of mTORC1 activation in a lysosomal nutrient sensing-dependent manner. In fact, amino acid depletion prevents mTORC1 recruitment and activation on the lysosomal membrane via AMPK-independent mechanisms, operated by the Rag GTPase complex, that recruits mTORC1, and by Rheb, that activates it (Lim and Zoncu, 2016). Once translocated on the lysosomal membrane, mTORC1 directly controls the subcellular localization of TFEB, a master autophagy regulator which continuously shuttles from the cytosol to the nucleus, where it mediates ATGs transcription and lysosomal biogenesis under starvation conditions (Sardiello et al., 2009; Settembre et al., 2011). mTORC1 phosphorylates TFEB in the nucleus and controls its export kinetics in a nutrient availability dependent manner (Napolitano et al., 2018). However, TFEB is able to promote autophagy gene expression not only during starvation but also during mitochondria depolarization (Nezich et al., 2015), ER stress (Martina et al., 2016) or lysosomal membrane permeabilization (Settembre et al., 2012).

CMA (Figure 1) is a one-by-one fashion-type of autophagy. About 30% of cytosolic proteins bear the Lys-Phe-Glu-Arg-Gln (KFERQ) sequence (Dice et al., 1986; Chiang and Dice, 1988; Chiang et al., 1989) which is recognized by cytosolic heat shock 70 kDa protein 8 (HSPA8, also known as hsc70). Then, hsc70, complexed with co-chaperones, shuttles the substrate proteins to the lysosomal membrane, where it binds to monomeric LAMP2A, a transmembrane receptor. This binding triggers LAMP2A multimerization into a translocation complex. Protein substrates must be unfolded by hsc70 and co-chaperones, and luminal hsc70 is also required to allow their translocation to the lysosomal lumen, where they are quickly hydrolyzed. Considering that the binding to LAMP2A is the rate-limiting step for CMA and that LAMP2A-deficient lysosomes are CMA-incompetent (Cuervo et al., 1997; Kaushik and Cuervo, 2012), LAMP2A is considered a marker of CMA. In particular, LAMP2A subcellular localization has been associated with Rab11 and Rab7 function (Zhang et al., 2017). The signaling pathways that mediate the upstream modulation of CMA are still not fully understood, however, a possible crosstalk between CMA and redox stress has been proposed via NFE2L2/NRF2 (nuclear factor, erythroid derived 2, like 2) (Pajares et al., 2018). Furthermore, oxidized proteins, such as Tau and α-syn are CMA substrates (Xilouri and Stefanis, 2015; Dikic, 2017).

Microautophagy (Figure 1) and endosomal microautophagy are the least characterized types of autophagy. Microautophagy is a constitutive active process by which cytoplasmic content is directly engulfed into lysosomes for degradation, without an intermediate autophagosome formation (Farre and Subramani, 2004). Instead, endosomal microautophagy occurs at the surface of late endosomes following the formation of multivesicular bodies (MVBs), that ultimately fuse with lysosomal membrane (Sahu et al., 2011; Uytterhoeven et al., 2015; Mukherjee et al., 2016; Oku et al., 2017). This process is responsible for maintaining the turnover of cellular nutrients via both a selective and hsc70-mediated mechanism or bulk degradation of proteins and organelles, such as mitochondria, peroxisomes and portions of nucleus (Olsvik et al., 2019). While microautophagy requires autophagic proteins participation, endosomal microautophagy is strongly associated with endosomal trafficking, which requires ESCRT complex that together with hsc70 mediates a more selective degradation (Sahu et al., 2011; Uytterhoeven et al., 2015; Mukherjee et al., 2016; Oku et al., 2017). Thus hsc70 exerts its function in both CMA and endosomal microautophagy, depending on the interactor protein.

Recently, different studies described another autophagic pathway, named “Precision Autophagy” (Kimura et al., 2015). Precision Autophagy is a receptor-regulator-mediated type of autophagy which requires factors that are part of the tripartite motif (TRIM) protein family that can act as both receptors and regulators of autophagy at the same time. Such proteins are capable of identifying their targets, even without the need of a tag, such as ubiquitin and galectins (Stolz et al., 2014). Those proteins exert their function by first recognizing exogenous and endogenous cargos through their C-terminal domain. After substrates recognition, TRIMs, acting as a receptor, stimulate autophagy by facilitating the assembly of autophagic machinery, primarily acting on ULK1, Beclin-1, ATG16L1 in their activated status. However, TRIMs-modulated autophagy can both enhance or reduce autophagic flux. In fact, TRIM28 can either positively modulate Beclin-1 activity through VPS34 activation (Yang et al., 2013) or mediate AMPK degradation acting as an E3 ligase (Pineda et al., 2015).

## Autophagy and Healthy Aging

Aging is a time-mediated physiological process which carries a decline in several molecular and cellular mechanisms contributing to cellular homeostasis. Defective proteostasis has been largely reported in a plethora of *in vitro* and *in vivo* aging models (Cuervo et al., 2005; Boland and Nixon, 2006; Balch et al., 2008; Ben-Zvi et al., 2009; Bingol, 2018; Boland et al., 2018). Bergamini et al. (2004) provided the first evidence that the efficiency of autophagy and, consequently, of detoxification mechanisms, worsen along with aging in liver tissue (Donati et al., 2001; Del Roso et al., 2003). Therefore, assessing the causes of autophagic dysfunction during senescence became fundamental in order to delay age-related phenotype in both physiological and pathological conditions. Autophagic decline has been considered as one of the major contributors to the age-dependent accumulation of dysfunctional organelles, cytoplasmatic content and misfolded/aberrant proteins that might combine to form potentially cytotoxic aggregates. Furthermore, failure in replacing old organelles, especially lysosomes, mitochondria and endoplasmic reticulum (ER), has important consequences on their morphology and functions, other than cellular homeostasis. In fact, one of the morphological features associated with senescence is the presence of enlarged lysosomes containing deposited lipofuscin, i.e., a pigment formed by a highly oxidized cross-linked protein aggregates, carbohydrates and lipids, remarkably resistant to proteolytic activity of lysosomal enzymes. Moreover, lipofuscin represents an important source of ROS via the Fenton reaction. Another common feature associated with ALP deficiency is the increase number of autophagic vesicles, detected by electron microscopic (EM) and immunofluorescence in different tissues (brain, heart, muscle and kidney). This scenario is exacerbated in postmitotic cells, such as neurons, where lipofuscin accumulation is even greater due to their reduced ability to dilute it through mitotic cycles (Rezzani et al., 2012).

Dysfunctional and damaged mitochondria are responsible for increased oxidative stress and free radical production (Rezzani et al., 2012). In 1956, Harman proposed “the free radical theory” according to which ROS damage cellular functions ensuing in the characteristic aging phenotype. In senescent cells, increased ROS production as well as reduced ROS cellular detoxification can damage mtDNA, causing an accumulation of mitochondrial mutations and hypoxia in senescent tissues, such as brain (Roberts et al., 1997), heart (Pepe, 2000), and kidney (Nangaku et al., 2008). Moreover, this age-related increase in oxidative stress may lead to enhanced protein oxidation, causing unfolding, exposure of hydrophobic residues and, finally, aggregation (Miquel, 1998; Hohn et al., 2017). In particular, ALP plays a more relevant role than UPS in promoting ROS detoxification. Indeed, chronic oxidative stress causes ATGs overexpression and mTORC1 inhibition, leading to an increased autophagic, but not proteasomal, activity (Chakraborty et al., 2019). Autophagy stimulation, e.g., by rapamycin treatment, can reverse increased ROS production, promoting neuronal survival (Ramirez-Moreno et al., 2019). Trehalose is another effective autophagy enhancer that can protect against oxidative stress in an mTORC1-independent manner. Trehalose is a naturally available disaccharide that facilitates the autophagic flux and exerts anti-oxidant effects by promoting the nuclear translocation of Nrf2, and the transcription of Nrf2 target genes (Mizunoe et al., 2018).

Aging has also been associated with activation of the immune response by several DAMPs, which are molecules capable of triggering the production of proinflammatory cytokines and chemokines, growth factors and components of extracellular matrix. The circular mtDNA and some mitochondrial proteins, like *N*-formyl peptides and cardiolipin, coming from senescent mitochondria, act as DAMPs through activating Toll-like receptor 9 signaling and formyl peptide receptor-1, respectively, causing an enhanced cytokine production from cultured monocytes (Pinti et al., 2014; Jang et al., 2018).

Impaired mitophagy is another common feature of aging (Sun et al., 2016). Mitophagy is a selective form of autophagy, since it regulates the removal of old and excessive mitochondria. Mitophagy starts with the recognition and targeting of damaged mitochondria, mediated by PINK1. PINK1 accumulates at the outer membrane of damaged mitochondria, where it recruits Parkin, an E3 ubiquitin ligase, which participates to mitochondria sequestration by autophagosomes (Jin and Youle, 2012). Age-related mitophagy decline is characterized by dysfunctional, fragmented and swollen mitochondria, which is accompanied by lower PINK1 expression in murine lung tissue (Sosulski et al., 2015) and aged cells (Terman et al., 2010).

Reduced clearance of damaged/dysfunctional organelles is not the only consequence of defective autophagy, since accumulation of long-lived, misfolded, oxidized proteins also occurs during aging. In fact, ALP, and to a lesser degree UPS are impaired in senescent tissues. LAMP1 and LAMP2A levels markedly decrease over the years resulting in a defective CMA activity. In aged cells, the decrease of LAMP2A levels is initially compensated by an increase of lysosome number (Cuervo and Dice, 2000; Ott et al., 2016). In senescence, however, the compensatory increase of the number of lysosomes and chaperones fails to further sustain CMA activity rate due to the increasingly small amount of LAMP2A. As a proposed mechanism, aging can affect either LAMP2A recycling from the lysosomal lumen or its stability at the lysosomal membrane. Indeed, lysosomal membrane accumulates cholesterol during aging, which results in an enhanced fluidity, and ultimately prevents LAMP2A multimerization (Rodriguez-Navarro et al., 2012). The increased quantity of cholesterol and ceramides can also create enlarged degradation membrane domains where LAMP2A accumulates and is degraded, which further reduces its abundance (Rodriguez-Navarro et al., 2012).

Likewise, autophagy decline is associated with a decrease in either autophagosomes formation or clearance (Bergamini and Signorini, 1991). It has been reported that the decreased autophagosomes formation might be due to reduced ATGs content, whereas the decrease of autophagosome clearance may be related to impaired fusion with the lysosomes and lysosomal activity (Kenessey et al., 1989; Sitte et al., 2000a, b).

Another contributor to impaired autophagy during aging is upregulation of mTOR pathway. In fact, mTORC1 inhibition has been widely investigated as a potential mechanism to slow down aging and promote life span extension. The first evidence of the role of autophagy in life-span expansion came from studies in *C. elegans*, where mutants of *daf-2*, a gene encoding insulin growth factor (IGF-1) receptor, showed reduced longevity due to the silencing of the autophagy gene *bec-1*, the ortholog of beclin-1. This suggested for the first time that enhancing autophagy would increase life-span (Melendez et al., 2003). Indeed, knocking down key autophagy proteins dramatically reduced the life-span of *daf-2* mutants (Melendez et al., 2003; Hars et al., 2007). The most effective methods to inhibit mTORC1 signaling in order to selectively activate autophagic machinery are caloric restriction (CR) and pharmacological blockage through administration of rapamycin or similar compounds (“rapalogs”) (Kapahi et al., 2010; Galluzzi et al., 2017). CR, a limiting food intake strategy without malnutrition, has been studied as a powerful anti-aging physiological intervention. CR-mediated autophagy enhancement requires activation of two energy sensors, like AMPK and Sirtuin 1, and inhibition of insulin/insulin-like growth factor (IGF) pathway, which, in turn, leads to downstream mTORC1 blockage. Abolition of high metabolic rate characterizing senescent cells is one of the accepted mechanisms by which reduced insulin/insulin-like growth factor or mTORC1 signaling promote longevity (Toth et al., 2008). Furthermore, mTORC1 plays a central role in the release of pro-inflammatory cytokines by old cells, a process named senescence-associated secretory phenotype (SASP) which is reversed by rapamycin treatment (Laberge et al., 2015). Moreover, in yeast, worms or flies CR is ineffective when mTORC1 pathway is already downregulated, demonstrating that a common signaling cascade underlies both strategies of life-span expansion (Grandison et al., 2009). Pharmacological blockade as well as genetic inhibition of mTORC1 increase life-span in several animal species, such as *C. elegans* (Vellai et al., 2003), *D. melanogaster* (Kapahi et al., 2004), *S. cereviasiae* (Kaeberlein et al., 2005), and mice (Harrison et al., 2009). On the contrary, ATG genes knockout or knockdown reverses life-span extension induced by rapamycin, suggesting that rapamycin selectively affects and enhances autophagosome formation. However, it is possible that other mechanisms come into play to extend the life-span, such as a reduced oxidative stress, as shown in rats (Moyse et al., 2019).

## Autophagy and Neurodegeneration

Autophagy is involved in maintaining proteostasis and general neuronal homeostasis, synaptic remodeling and activity, axo-dendritic plasticity and mitochondrial clearance in CNS. Both clinical studies and preclinical models described neuronal atrophy, reduced cargo amount, impaired autophagy and mitophagy in age-related diseases (Boland and Nixon, 2006; Menzies et al., 2017; Boland et al., 2018). To further investigate the specific role of autophagy in neuronal cell-types, several *Atg5* and *Atg7* KO models have been developed (Galluzzi et al., 2017; Boland et al., 2018). Those studies provided evidence that autophagy is essential for axonal homeostasis and that each neuronal cell type responds differently to autophagy decline due to a diverse susceptibility to cytotoxic proteins aggregation (Tsvetkov et al., 2013). *Atg5* and *Atg7* KO mice showed early onset neurodegeneration, while beclin-1 knockdown increased susceptibility of hippocampal neurons to energy deprivation (Fekadu and Rami, 2016). Furthermore, selective and conditional genetic deletion, via Nestin-Cre technology, of *Atg5* and *Atg7* causes defective autophagy and consequent accumulation of intracellular aggregation-prone proteins in neurons and glia (Hara et al., 2006; Komatsu et al., 2006a, 2007b). Although the aged brain is accompanied by a deficiency in both general and selective types of autophagy (see above), an increased compensatory mitophagy in the later phase of neurodegenerative diseases has been observed. In fact, beyond ATP production, mitochondria are also involved in protein clearance, as proven by the intake of aggregation-prone proteins, such as amyloid-β, superoxide dismutase 1 (SOD1) variants and α-syn. Nevertheless, this compensatory process leads to a higher chance of developing mitochondria damage due to increase exposure to cytotoxic proteins, like α-syn. Therefore, enhancing PINK1-Parkin-driven mitophagy has been viewed as a strategy to improve both mitochondria quality control and protein degradation (Hertz et al., 2013; Georgakopoulos et al., 2017).

Neurodegenerative disorders of aging, such as AD, PD, and HD, have as a common feature the formation of cytotoxic aggregates. Those aggregates are made of proteins that can be oxidized, wrongly processed, misfolded or cross-linked by cellular post-translational machinery. These proteins are considered “toxic” because they lose their physiological conformation and functions, alter cellular trafficking, and block CMA or UPS, promoting their spreading throughout the CNS in a prion-like manner (Brundin et al., 2010; Brundin and Melki, 2017). Moreover, neurons are postmitotic cells, thus are not capable of diluting protein aggregates through mitotic cycles, making cells even more susceptible to their potential toxicity properties. In fact, neurons activate different compensatory mechanisms to reduce protein accumulation, for instance sequestering them in hydrophobic agglomerates or combining them with microtubules to form aggresomes (Ciechanover and Kwon, 2017). Even though this strategy can be neuroprotective in the early stages of age-related diseases, it fails to handle the increased cytotoxic burden in the later stages. As reported above, autophagy declines physiologically with senescence. In the context of neurodegenerative diseases, this age-dependent decline appears to be exacerbated because it is accompanied by the impairment of ALP machinery due to the accumulation of neurotoxic proteins. Furthermore, in most cases, neurodegenerative diseases are associated with the inheritance of mutations in genes controlling the autophagic process, suggesting that aberrant autophagy might contribute to neurodegeneration (Ravikumar et al., 2002; Berger et al., 2006; Ravikumar et al., 2006).

### Autophagy and Parkinson’s Disease

The neuropathological hallmarks of PD are the loss of dopamine (DA) neurons in the SNpc and the accumulation of intracellular aggregates containing α-syn, named LBs. Changes in ALP markers were reported in the human brain which are consistent with an impairment of autophagic machinery. A significant reduction of lysosomal and CMA markers, such as LAMP1, LAMP2A, Hsc70 or Cathepsin D, associated with an increase of autophagic markers LC3II and p62 levels, was reported in the whole-brain (Mamais et al., 2018) or SNpc (Chu et al., 2009; Dehay et al., 2010) of idiopathic PD patients (Xilouri and Stefanis, 2015). Moreover, LC3II has been shown to colocalize with α-syn in LBs (Alvarez-Erviti et al., 2010; Dehay et al., 2010). The patterns of ALP markers were compared in the brains of idiopathic vs. G2019S LRRK2 PD patients (Mamais et al., 2018). Interestingly, G2019S PD patients did not show the increase of LC3II, p62 and ULK-1 levels observed in idiopathic PD patients, but instead a significant reduction of LAMP1 levels. This was interpreted as being due to a different pathobiology associated with idiopathic and G2019S LRRK2 PD (Mamais et al., 2018). Consistent with this view, G2019S LRRK2 cases had lower insoluble α-syn levels compared to idiopathic PD patients, suggesting differences in the biochemical properties of aggregated α-syn (Mamais et al., 2013). In another study, an increase of LAMP2A but not LAMP1 levels was reported in the cholinergic cells of the motor nucleus of the vagal nerve in G2019S LRRK2 patients compared to non-neurological controls, which was explained as being due to either a compensation for impaired CMA or a chronic reduction in LAMP2A turnover (Orenstein et al., 2013). ALP changes were also investigated in the brains of patients suffering from dementia with LB (DLB), another type of synucleinopathy, in comparison with brains of AD patients (Crews et al., 2010; Higashi et al., 2011). In the first study, an increase of mTOR along with a reduction of ATG7 (but not beclin-1, ATG5 or ATG12) was detected in DLB vs. AD brains (Crews et al., 2010) whereas in the latter study, an increase of LC3II was observed in DLB but not AD patients, a reduction of LAMP2 levels being common (Higashi et al., 2011). These two studies suggest different roles of autophagy in these diseases.

Studies on mitophagy in PD were the first ones to elucidate the crucial role of autophagy in age-related degeneration in CNS. In fact, blocking mitochondrial complex I by administration of low doses of MPP+ is one of the most common strategies to study PD pathogenesis (Zhu et al., 2012). Early onset autosomal recessive forms of PD, also have been related to mutations in genes that encode PINK1 (Valente et al., 2004) and the E3 ubiquitin ligase Parkin (Kitada et al., 1998), both involved in recognition, targeting and degradation of damaged mitochondria. Although no behavioral changes have been observed in *Parkin*^–/–^ mice (Goldberg et al., 2003; Itier et al., 2003; Perez and Palmiter, 2005), disrupted mitochondrial function, response to DA and synaptic plasticity occur in the striatum (Palacino et al., 2004; Kitada et al., 2009). Even *Pink1* deletion results in impaired mitochondrial function and increased susceptibility to oxidative stress in mice (Gautier et al., 2008). Enhancing PINK1 activity has been reported to rescue from apoptotic signals, representing a potential strategy for idiopathic PD (Petit et al., 2005; Pridgeon et al., 2007; Klinkenberg et al., 2010). Accordingly, administration of kinetin, a precursor of an ATP analog, increases PINK1 function as proven by a more abundant phosphorylated form of Bcl-xL, a protein that mediates mitochondrial-induced apoptosis (Adams and Cory, 1998; Gross et al., 1999). Moreover, kinetin can promote PINK1-dependent recruitment of Parkin on the outer depolarized mitochondrial membrane allowing mitophagy to start (Hertz et al., 2013). Therefore, restoring mitophagy rate might be a new neuroprotective strategy to reduce DA neuron degeneration typical of PD (Park et al., 2017).

There is evidence for a causative relationship between CMA dysfunction and intracellular accumulation of α-syn, a common feature of PD. Indeed, α-syn is a qualified substrate for CMA as it bears a KFERQ-like motif. Downregulating key components of CMA pathway, such as LAMP2A in rat midbrain (Xilouri et al., 2016) and Hsc70 in neuronal cell models (Sala et al., 2016), leads to increase of α-syn intracellular content, neural loss and behavioral deficits in rats. Furthermore, either stress-mediated or PD-linked A30P and A53T mutations in the α-syn gene (SNCA) determine the production of aberrant α-syn which displays a fivefold higher affinity for LAMP2A compared to the WT protein, preventing LAMP2A translocation across the lysosomal membrane and degradation of CMA targets (Cuervo et al., 2004; Martinez-Vicente et al., 2008). Mutations in GBA, which encodes the lysosomal enzyme GCase, are major risk genetic factors for PD, since they lead to a protein loss-of-function and lysosomal dysfunction. Mutations in GBA are major genetic factors for PD, encoding the lysosomal enzyme GCase, which leads to a protein loss-of-function and lysosomal dysfunction. The GBA1 homozygous mutations, instead, cause a lysosomal storage disorder, named Gaucher Disease which is characterized by excessive accumulation of glucosylceramide, a GCase substrate, into the lysosomal lumen, with subsequent autophagy impairment. The relevance of this pathway is further strengthened by the finding that even PD patients without GBA1 mutations showed a reduced GCase activity in the same cerebral areas displaying α-syn deposition (Mazzulli et al., 2011; Murphy et al., 2014). Besides, it has been reported that chronic pharmacological blockade of GCase in A53T α-syn transgenic mice further promoted exosome-associated α-syn accumulation and secretion. This supported the view that GCase directly regulates α-syn extracellular homeostasis and might be studied as a new therapeutic target in PD (Papadopoulos et al., 2018).

## LRRK2 and Autophagy

LRRK2 is a large multidomain protein with central GTPase Ras-of-Complex (ROC) and kinase domains, surrounded by protein-protein interaction domains (Mata et al., 2006; Cookson, 2010; Mills et al., 2014). LRRK2 mutations are the most common genetic cause of familial PD (Zimprich et al., 2004; Paisan-Ruiz et al., 2005) and GWAS studies revealed LRRK2 represents a risk factor for idiopathic PD (Nalls et al., 2014). At least six pathogenic mutations of LRRK2 have been identified: two in the kinase domain (G2019S and I2020T), three (R1441C/G/H) in the GTPase/ROC domain, and one (Y1669C) in the CoR domain (Reed et al., 2019). The G2019S mutation is most frequently associated with PD, followed by R1441C/G/H (Gasser, 2009; Okubadejo et al., 2018). LRRK2-associated PD is clinically and neuropathologically indistinguishable from idiopathic PD, most cases, particularly G2019S cases, presenting with nigrostriatal dopaminergic degeneration and LBs (Marras et al., 2011; Kalia et al., 2015). The penetrance of LRRK2-mediated PD is age-dependent but incomplete. LRRK2 mutations facilitate PD through several possible mechanisms, since LRRK2 is involved in a multitude of cellular functions and pathways, among which vesicle trafficking, cytoskeletal dynamics, neurotransmitter release, synaptic plasticity, Golgi and mitochondrial function, and immune response. In addition, several studies have pointed out the role of LRRK2 in autophagy (Roosen and Cookson, 2016; Manzoni and Lewis, 2017), which will be covered in the next chapters.

### *In vitro* Studies

The first indirect evidence of LRRK2 modulation of proteostasis came from the 2006 study of MacLeod et al. (2006) showing that primary cortical neurons overexpressing G2019S or I2020T LRRK2 had tau-positive aggregates, MVB accumulation and swollen lysosomes (Table 1). That LRRK2 modulates autophagy was shown for the first time by Plowey et al. (2008) in SH-SY5Y neuroblastoma cells overexpressing WT LRRK2, G2019S LRRK2 (a kinase-enhancing mutation) (West et al., 2005; Greggio et al., 2006), or K1906M LRRK2, a kinase dead (KD) mutation (Table 1). Cells overexpressing G2019S LRRK2 displayed neurite shortening, together with an increase in the number and size of LC3-reactive autophagic vacuoles (AV), both at the neuritic and the somatic levels. The autophagic inducer, rapamycin, further enhanced these effects whereas blocking autophagy through LC3 or *Atg*7 knock-down (with RNA-interference) caused their reversal, suggesting that G2019S overexpression is associated with enhanced autophagy and neurite shortening. It is worth noting that in this model, neither WT LRRK2 nor K1906M LRRK2 overexpression altered the autophagic flux, suggesting that not the loss but the increase of kinase activity might be instrumental to LRRK2 neurotoxic effect. Other studies confirmed that G2019S or R1441C LRRK2 mutants are associated with an increased autophagy (Gomez-Suaga et al., 2012b; Bravo-San Pedro et al., 2013; Orenstein et al., 2013; Su and Qi, 2013; Yakhine-Diop et al., 2014; Su et al., 2015). Nonetheless, others *in vitro* studies in different cell lines showed that LRRK2 G2019S and/or R1441C overexpression leads to inhibition of autophagy (Alegre-Abarrategui et al., 2009; Sanchez-Danes et al., 2012; Manzoni et al., 2013a, 2016; Wallings et al., 2019). A possible difference between mutations in the Roc-COR and kinase domains in terms of response to starvation-induced autophagy was also reported (Manzoni et al., 2013b). G2019S LRRK2 overexpression was also found to inhibit specific forms of autophagy such as CMA (Orenstein et al., 2013) or mitophagy (Wauters et al., 2019); in these studies, autophagy was found to be increased (possibly to compensate for CMA blockade) (Orenstein et al., 2013) or to be unchanged (Wauters et al., 2019). The role of the kinase activity of LRRK2 was investigated using LRRK2 kinase inhibitors. LRRK2-IN1, GSK2578215A and CZC25146 were reported to increase (Manzoni et al., 2013a, 2016) or to inhibit (Saez-Atienzar et al., 2014; Schapansky et al., 2014) the autophagic flux *in vitro.* Unfortunately, first generation inhibitors were employed in these studies, which are characterized by low kinase specificity and off-target effects (West, 2017). Interestingly, however, more selective LRRK2 inhibitors MLi-2 and PF-06447475 increased the autophagy flux in primary cortical neurons obtained from BAC G2019S (but not R1441C) mice causing the opposite effect in non-transgenic cultures (Wallings et al., 2019). The issue of whether LRRK2 acts as negative or positive modulator of autophagy is also still unclear (Table 1). Neurons or macrophages obtained from LRRK2 KO mice show an increase of LC3II levels and autophagic flux, suggesting that endogenous LRRK2 inhibits autophagy (Manzoni et al., 2016; Hartlova et al., 2018; Wallings et al., 2019). However, most studies reported that WT LRRK2 overexpression increased autophagy (Gomez-Suaga et al., 2012b; Orenstein et al., 2013; Su and Qi, 2013; Roosen and Cookson, 2016), with one study (Plowey et al., 2008) reporting no effect and, another (Wallings et al., 2019), inhibition. Acute knock-down of LRRK2 was shown to be ineffective (Orenstein et al., 2013; Manzoni et al., 2016), to increase (Alegre-Abarrategui et al., 2009) or to inhibit (Schapansky et al., 2014) autophagy. Therefore, not only the regulation of autophagy operated by LRRK2 is mechanistically complex, but also experimental outcomes seem to be affected by a number of variables including the cell model, expression levels of LRRK2, LRRK2 kinase inhibitor used, autophagic markers investigated and protocols adopted (Klionsky et al., 2016). In this respect, it should be noted that although autophagy was monitored through quantification of LC3II levels, not all studies investigated the autophagic flux, i.e., fold-changes following application of autophagy inhibitors, which would allow to conclude whether the increase of LC3II levels is due to an increase of autophagosome synthesis/maturation or a blockage of their clearance.

**TABLE 1:** Synopsis of the *in vitro* studies investigating the impact of genetic and pharmacological manipulation of LRRK2 on autophagy and its markers.

	**Cell type**	**LRRK2 manipulation**	**Autophagic marker**	**Phenotype**
MacLeod et al., 2006	Primary cortical neurons	G2019S, I2020T OE	Swollen lys, vacuolized mitochondria, phospho-tau-positive aggregates, ↑ MVB	Neurite shortening
Plowey et al., 2008	SH-SY5Y	G2019S OE	↑ LC3 puncta, ↑ AV number and size ↑ autophagy	Neurite shortening, potentiated by rapamycin and reversed by LC3 or Atg7 kd but not 3-MA. Blocked by MEK-inhibitor
		WT, K1906M OE (KD)	↔	
Alegre-Abarrategui et al., 2009	HEK293T, VERO	R1441C, G2019S OE	↑ LC3 puncta, ↑ MVB and AV ↓ autophagy	Colocalization of LRRK2 with p62 and LC3 in MVB and AV, skein-like inclusions in R1441C transfected cells
		LRRK2 kd	↓ LC3II levels, ↑ LC3 turnover ↑ autophagy	Protects against BFA-induced cell death in starvation conditions
Gomez-Suaga et al., 2012b	HEK293T, PC12	WT, G2019S OE	↑ LC3 puncta, ↑ LC3II levels ↑ mature lys ↑ autophagic structures ↑ p62, ↑ autophagy	NAADP-dependent, Ca^++^/CaMKK/AMPK-dependent. Increase cell-death triggered by an UPS inhibitor (rescued by rapamycin)
		K1906M OE (KD)	↔	
Sanchez-Danes et al., 2012	Human-derived iPSC	G2019S	↑ LC3 puncta ↑ lipid droplets, ↑ p62, ↓ LC3 flux, ↓ AV ↓ autophagic clearance	Shorter/fewer neurites
Orenstein et al., 2013	SH-SY5Y, HEK293T, human-derived iPSC, primary neurons	WT OE	↑ LAMP2A	Impaired CMA with compensatory ↑ autophagy
		G2019S OE	↑ LAMP2A ↔ LC3	
		LRRK2 kd	↔	
Bravo-San Pedro et al., 2013	Human fibroblasts	G2019S	↑ autophagic flux, ↑ AP, ↓ p62 ↑ LAMP2A, LC3, beclin-1	Inhibited by MAPK1/3 inhibitor
Manzoni et al., 2013a	H4 (neuroglioma), primary astrocytes	LRRK2 kinase inhibition: LRRK2-IN-1, GSK2578215A CZC-25146	↑ LC3I, LC3II ↑ p62 ↑ autophagic flux	mTOR-independent
Manzoni et al., 2013b	Human fibroblasts	G2019S, R1441G, Y1699C	↓ LC3II/LC3I after starvation, ↓ p62-positive in LRRK2 Y1699C and R1441G cells, ↔ p62, LAMP1 levels	mTOR-independent
Su and Qi, 2013	HEK293T	WT, G2019S OE	↑ LC3II ↑ autophagy	Drp1-dependent mitochondrial fragmentation autophagy
Saez-Atienzar et al., 2014	SH-SY5Y	LRRK2 kd LRRK2 kinase inhibition: LRRK2-IN-1, GSK2578215A	↑ LC3II, p62 ↑ autophagy ↓ AP-lys fusion ↓ autophagic flux	Induces mitochondrial fission and cell-death. Exacerbated by autophagy inhibitors
Schapansky et al., 2014	RAW264.7 (macrophages), BV2 (microglia)	LRRK2 kd, LRRK2 kinase inhibition: LRRK2-IN-1, GSK2578215A	↓ autophagic flux ↓ LC3II	↓ clearance of Q74 protein aggregates in microglial cells
Yakhine-Diop et al., 2014	Human fibroblasts	G2019S	↑ LC3II, beclin-1, LAMP2A, Cath B, Swollen lysosomes, ↑ autophagy	↑ MPP+-induced cell death, MPP+-dependent mTOR dephosphorylation
Su et al., 2015	Human fibroblasts	G2019S	↑ autophagic flux	Mitochondrial depolarization and mass loss
	HeLa	WT, G2019S OE	↑ p62 translocation to mitochondria, ↑Bcl-2 ↑ mitophagy	Bcl-2-dependent mitophagy
Manzoni et al., 2016	H4	LRRK2 kinase inhibition: LRRK2-IN-1, GSK2578215A	↑ LC3II ↔ pULK1 ↔ pS6K	mTOR/ULK1-independent, PI3P and Beclin-1-dependent
		LRRK2 kd	↔	
	Primary astrocytes	LRRK2 KO	↑ LC3II	
Schapansky et al., 2018	Primary cortical neurons	G2019S	↓ LAMP1, LC3I ↓ autophagic flux ↑ lys pH	↑ tau phosphorylation ↑α-syn aggregates
		LRRK2 kinase inhibition: GSK2578215A, CZC25146	↔	Rescues lys changes and reduces α-syn aggregates
Hartlova et al., 2018	Bone marrow-derived macrophages	LRRK2 kinase inhibition: GSK2578215A	↑ LC3II, ↓ p62	BFA-insensitive
		LRRK2 KO	As above	
Wallings et al., 2019	Primary rat cortical neurons	R1441C	↑ LC3II, ↔ p62, LAMP1 ↓ autophagic flux, ↓ AP-lys fusion ↓ lys protein degradation ↑ lys pH	Unresponsive to LRRK2 kinase inhibition (MLi-2, PF06447475)
		WT, G2019S	↓ autophagic flux	Reversed by LRRK2 kinase inhibition
		Non transgenic	↓ autophagic flux after LRRK2 kinase inhibitors	
		LRRK2 KO	↑ LC3II, ↔ p62, LAMP1 ↑ lys protein degradation	
Ho et al., 2019	MEF, primary neurons	R1441G	↓ lys activity ↓ LAMP2A, ↓ CMA ↔ autophagy	↓α-syn clearance Effects rescued by CMA-activator
Wauters et al., 2019	Human fibroblasts	G2019S, R1441C	↔ LC3II ↔ autophagy	Rab10-dependent impairment of mitophagy
Bonello et al., 2019	COS7 Human fibroblasts	G2019S, R1441C	N/A	Impairs Pink1/parkin-mediated mitophagy
		D1994A (KD), LRRK2 kinase inhibition: LRRK2-IN-1		Rescues G2019S LRRK2 effects

*↑ increase, ↓ decrease, ↔ no change; A, autophagosome; AV, autophagic vacuoles; BFA, bafilomycin A1; kd, knock-down; KD, kinase dead; KO, knock-out; Lys, lysosomes/lysosomal; MEF, mouse embryonic fibroblasts; MVB, multi-vesicular bodies; OE, overexpression; vmDA, ventral midbrain dopamine.*

### *In vivo* Studies

#### Invertebrates

The first published evidence that LRRK2 affects autophagy *in vivo* was produced in yeasts where administration of fragments of human LRRK2 carrying mutations that impair GTPase activity caused neuronal death, vesicle trafficking defects and AV accumulation (Xiong et al., 2010) (Table 2). In *Drosophila melanogaster* (Imai et al., 2008) pointed out that LRRK2 modulates protein synthesis through phosphorylation of eIF4E binding protein (4E-BP), which lies along the mTOR pathway, suggesting that LRRK2 modulates ALP. Overexpression of WT or I2020T LRRK2 hyperphosphorylated 4E-BP, which resulted in reduced 4E-BP binding to eIF4E, and deregulation of protein translation. Interestingly, however, no changes in levels or phosphorylation of mTOR were found, questioning the role of autophagy in these events (Imai et al., 2008). Later study in *Drosophila melanogaster* more convincingly proved the role of autophagy in LRRK2 toxicity, showing that activation of autophagy via pharmacological or genetic stimulation of AMPK protected against mitochondrial toxicity and DA cell loss induced by expression of human G2019S LRRK2 (Ng et al., 2012). ALP defects were also observed in follicle cells of *Drosophila melanogaster*, where the *LRRK2* homolog *Lrrk* colocalizes with, and binds to Rab7 and Lamp1 in late endosomes and lysosomes, and to a much lesser extent to Rab5 in early endosomes (Dodson et al., 2012, 2014). *Lrrk* null flies showed enlarged Rab7-positive structures containing undigested cytosolic material, increased autophagosomes and enlarged early endosomes containing monoubiquitinated proteins, consistent with the view that loss of *Lrrk* function blocks the late endosomal to lysosomal maturation, impairs the degradative properties of lysosomes and the substrate delivery to lysosomes. Interestingly, this phenotype was reversed by overexpression of WT Lrrk or G1914S Lrrk (a mutant with enhanced kinase activity equivalent to G2019S LRRK2) (Dodson et al., 2014). Studies in *C. elegans* also demonstrated that LRRK2 regulates autophagy. Saha et al. (2015) using a fluorescence gene reporter fused with lgg-1, the LC3 homolog, revealed that aging impairs the autophagic flux: hG2019S or hR1441G LRRK2 worsened this effect whereas WT LRRK2 and a LRRK2 KD mutant improved it. Nonetheless, when co-expressed with α-syn, both G2019S LRRK2 and, to a lesser extent, WT LRRK2, impaired autophagy in an age-dependent way, which was associated with a greater loss of DA neurons in older nematodes (Saha et al., 2015). Interestingly, a minimal effect of G2019S LRRK2 on the expression of the LAMP1 homolog, lmp-1, was detected, suggesting that, at variance with *Drosophila*, LRRK2 minimally altered lysosomal function (Saha et al., 2015).

**TABLE 2:** Changes in autosomal-lysosomal pathways in LRRK2 KO mice and rats *in vivo*.

	**Strain age (mos)**	**Brain**	**Kidney**	**α-syn levels**	**Note**
Tong et al., 2010	LRRK2 KO mice (20 m)	No accumulation of α-syn or ub	↓ UPS activity ↑ LC3I, ↓ LC3II ↑ p62	↑α-syn aggregation ↑ pS129 αsyn	Age-dependent
Herzig et al., 2011	LRRK2 KO mice (14 m)	N/A	↑ Akt, TSC2, mTOR, 4E-BP1 and pT37/46 4EBP1, ↑ p62 **↔** pT308 Akt, S6, LC3I/II	**↔**	Mild ↑ lamellar bodies in lung type II pneumocytes
Tong et al., 2012	LRRK2 KO mice (7 m)	N/A	↓ LC3I, ↑ LC3II ↓ p62 ↑ LAMP1, LAMP2A, CathB and D	↓α-syn insoluble fraction	Biphasic
	LRRK2 KO mice (20 m)		↑ LC3-I, LC3II ↑ LAMP1, LAMP2A, CathB and D	↑α-syn soluble and insoluble fraction	
Hinkle et al., 2012	LRRK2 KO mice (12 m,18 m)	N/A	↑ p62	**↔**	
Baptista et al., 2013	LRRK2 KO rats (1–12 m)	N/A	↑ LAMP1, LAMP2A	N/A	Age-dependent
Fuji et al., 2015	LRRK2 KO mice (2–3 m)	N/A	↑ lysosomes	N/A	↑ lamellar bodies in lung type II pneumocytes
Giaime et al., 2017	LRRK KO^∗^ mice (15 m)	↑α-syn or ub ↑ p62, ↓ LC3I, ↑ LC3II	N/A	↑α-syn and HMW α-syn in striatum and SNpc but not CCx	Age-dependent

*^∗^Mice lacking both LRRK1 and LRRK2, i.e., double KO mice. ↑ increase, ↓ decrease, **↔** no change; AP, autophagosome; Cath, cathepsin; CCx, cerebral cortex; HMW, high molecular weight; SNpc, substantia nigra pars compacta; ub, ubiquitinated proteins.*

#### Rodents

Original study in LRRK2 KO mice failed to find brain pathology (nigrostriatal degeneration and α-syn or ubiquitinated protein accumulation) and changes in brain autophagy markers in 2-year-old LRRK2 KO mice (Tong et al., 2010) (Table 2). Nonetheless, this study unraveled that loss of LRRK2 caused striking age-dependent pathology selectively in the kidney, which expresses the highest levels of LRRK2 in the mouse (sixfold greater than in the brain). An increase of both soluble and insoluble forms of α-syn, along with the levels of pSer129-α-syn, were found at 20 months but not at 10 weeks of age. Since α-syn is degraded by ALP and UPS, this pointed to an impairment of α-syn clearance in aged kidneys. An impairment of UPS and ALP due to the deletion of LRRK2 was further suggested by the accumulation of ubiquitinated high-molecular weight proteins and lipofuscin granules, and by the analysis of the autophagic flux, which revealed mild elevation of LC3I, dramatic reduction of LC3II levels, and compensatory accumulation of p62.

The same groups of authors later observed (Tong et al., 2012) that changes in ALP function were actually biphasic. At 7 months, a reduction of LC3I/LC3II ratio and p62 levels was associated with a reduction of insoluble α-syn aggregates (soluble α-syn levels were undetectable), indicating an increase of ALP activity, whereas at 20 months an increase of LC3I/LC3II ratio and p62 levels was associated with elevation of both soluble and insoluble α-syn levels, indicating normalization of autophagy. Analysis of lysosomal proteins and proteases at key ages did not reveal qualitative age-dependent changes since levels of LAMP1 and LAMP2 as well as cathepsin B were found to be elevated already from the first month of age (significant elevation of cathepsin D was observed at both 7 and 20 months). However, EM analysis revealed kidney-specific age-related accumulation of autolysosomes and lipofuscin granules confirming that loss of LRRK2 is associated with lysosomes alterations. Kidney pathology in LRRK2 KO mice was substantially confirmed by Hinkle et al. (2012) and Fuji et al. (2015). These authors analyzed kidneys at 3, 12, and 18 months. Pigmentation and lipofuscin staining, already evident at 3 months, worsened with aging. p62 levels progressively increased with age, at variance with WT controls where they appeared at 12 months. Different from previous studies, however, no changes in α-syn staining was observed in these mice. Also at variance with previous data, elevated LC3II were detected in 18-month-old LRRK2 KO mice, with no difference observed at younger ages (Hinkle et al., 2012). Likewise, LRRK2 KO rats (Baptista et al., 2013) showed striking age-dependent kidney pathology, with lipofuscin accumulation and irregular hyaline droplets accumulation in kidney tubular epithelium, starting at 4 months. In the same cells, progressive increase of immunohistochemical staining for LAMP1 and LAMP2 was noted, which was overall interpreted as an impairment of lysosomal function.

Novartis group analyzed in great detail autophagy in 14-month-old LRRK2 KO mice in comparison with 5-month-old G2019S KI mice, which show enhanced kinase activity *in vivo* (Yue et al., 2015; Longo et al., 2017; Mercatelli et al., 2019), and 6-month-old KD mice, which instead show no kinase activity *in vivo* (Herzig et al., 2011; Mercatelli et al., 2019). Microvacuolization in tubular epithelial cells of LRRK2 KO mice appeared already at 1.5 months, and progressively worsened over time, with lipofuscin-like pigments appearing at 8 months. Similar changes were observed for the first time also in type II pneumocytes of lungs, other organs (brain, spleen, liver, heart) being spared, indicating a crucial role for LRRK2 in kidney and lung homeostasis. A similar pathology was observed in the kidneys of KD, but not G2019S KI, mice, consistent with the view that loss of LRRK2 or its kinase activity causes derangement of kidney homeostasis (Herzig et al., 2011). In line with this, LAMP1 or LAMP2 immunohistochemical staining was elevated in 6–9-month-old LRRK2 KO or KD mice, respectively. EM analysis revealed progressive increase in number and size of secondary lysosomes in LRRK2 KO mice, again confirming alteration in lysosome homeostasis. In keeping with Hinkle et al. (2012) and in contrast with Tong et al. (2010, 2012), no changes in α-syn levels were observed in the kidney of these mice. This study confirmed the elevation of p62 levels in the LRRK2 KO kidney (Tong et al., 2010), and compared for the first time a wider panel of autophagic and lysosomal markers using Western analysis. The resulting picture, however, was quite confusing, since the Akt/mTOR pathway was perturbed in all genotypes, but with different patterns. Akt levels were elevated in KD and LRRK2 KO mice, but not G2019S KI mice. However, TSC2 and mTOR levels were reduced in KD and elevated in LRRK2 KO and G2019S KI mice. Perhaps consistent with an overactivation of the Akt/mTOR pathway with consequent autophagy inhibition, levels of the mTOR-regulated translation initiation factor 4E-BP1 and its phosphorylated Thr37/46 forms were also elevated in LRRK2 KO mice whereas no changes were observed in G2019S KI mice. S6 kinase levels were unchanged in all genotypes. So were LC3I and LC3II levels in LRRK2 KO and KD mice (not investigated in G2019S KI mice). Kidney and lung pathology were also confirmed in 10–12 weeks LRRK2 KO mice by a Genentech study aimed at assessing the safety of two LRRK2 inhibitors (GNE-7915 and GNE-0877) (Fuji et al., 2015). This study also revealed that administration of LRRK2 kinase inhibitors did not cause vacuolization, i.e., lysosome dysregulation, in rodent tissues. At variance with rodents, however, an increase of lamellar bodies in type II pneumocytes of the lung of non-human primates, similar to those observed in LRRK2 KO mice, was shown (Fuji et al., 2015), which might be consistent with the view that inhibition of LRRK2 kinase activity impairs autophagy. The different species sensitivity was attributed to different residual LRRK2 levels after LRRK2 inhibition (Fuji et al., 2015). It therefore appears that removal of LRRK2 impairs ALP machinery in kidney and pneumocytes. Nonetheless, subtle changes observed in G2019S KI mice would not allow to rule out that also an increase of LRRK2 kinase activity might perturb autophagy, possibly indicating that ALP is physiologically regulated within a narrow range of LRRK2 kinase activity.

What also clearly emerged from these studies is that genetic deletion of LRRK2 does not impact autophagy in the brain. A more recent study, however, revealed that the functional homolog of LRRK2, i.e., LRRK1, might play a compensatory role in LRRK2 KO mice (Giaime et al., 2017). Indeed, although any changes of autophagic markers were found in mice constitutively lacking either LRRK2 or LRRK1, double KO mice showed age-dependent increase of p62 and LC3II levels, reduction of LC3I levels, and AV accumulation, indicating an impairment of autophagy (Giaime et al., 2017). Development of a selective antibody might help elucidate the role of LRRK1 in autophagy and, more in general, in cellular homeostasis.

Following the report of tau hyperphosphorylation in brain lysates of 9–10-month-old LRRK2 BAC R1441C mice (Li et al., 2009), the impact of LRRK2 mutants (i.e., LRRK2 G2019S or R1441C/G) on brain autophagy was widely investigated (Table 3). Autophagy abnormalities were detected in the striatum of 17–18-month-old hG2019S transgenic mice (enlarged AV and increased autophagosomes, associated with aggregated and damaged mitochondria) (Ramonet et al., 2011). Autophagy changes consistent with reduced autophagic flux were also revealed in G2019S KI mice. Specifically, 15-month-old G2019S KI mice showed an increase of LC3II levels in a whole brain lysate (Yue et al., 2015) whereas 20-month-old G2019S KI mice, showed a reduction of LAMP1 in the cerebral cortex (Schapansky et al., 2018). This was confirmed by experiments in primary cortical neurons obtained from G2019S KI mice, where autophagy impairment was reversed by LRRK2 inhibitors (Schapansky et al., 2018). Moreover, 12–19-month-old G2019S overexpressing mice showed accumulation of LC3 and p62 levels along with levels of GRB78/BiP, an ER stress marker (Ho et al., 2018). In this study, it was shown that LRRK2 phosphorylates leucyl-tRNA synthetase, a regulator of protein translation and mTOR interactor, and that G2019S LRRK2 is associated with ER stress, accumulation of α-syn aggregates and autophagic markers. It was therefore proposed that inhibition of autophagy by G2019S LRRK2 would be secondary to ER stress and misfolded protein engulfment (Ho et al., 2018). Possibly in general agreement with previous studies, 22-month-old BAC G2019S and R1441C mice showed an increase in LC3 puncta in SNpc DA neurons, as measured by immunohistochemistry (Wallings et al., 2019). However, considering the low expression levels of LRRK2 in mouse nigral DA neurons (Biskup et al., 2006; Simon-Sanchez et al., 2006; Taymans et al., 2006; Melrose et al., 2007; West et al., 2014), it is not clear whether these effects are truly LRRK2-dependent and, if so, if they rely on LRRK2 kinase activity or scaffold properties of LRRK2.

**TABLE 3:** Changes in autosomal-lysosomal pathways in LRRK2 mutant mice and rats *in vivo.*

	**Model**	**Age (mos)**	**Brain**				**Kidney**
				***α-syn***	***Area***	***NDG***	
Li et al., 2009	BAC R1441C mice	9–10	↑ phospho-tau	N/A	Whole brain	No	N/A
Ramonet et al., 2011	G2019S TG mice (PDGF prom)	19–20	Enlarged vacuoles, ↑ AP Mitochondrial damage	N/A	CCx	18%	N/A
	R1441C TG mice (PDGF prom)	23–24	As above but milder			No	
Herzig et al., 2011	G2019S KI mice	5	N/A	No	N/A	No	↑ TSC2, mTOR **↔** Akt, 4E-BP1 and pT37/46 4E-BP1
	Kinase-dead (D1994S KI mice)	6	N/A	No	N/A	No	↑ Akt, 4E-BP1 ↓ TSC2, mTOR **↔** pT308 Akt, S6K and pS235/236 S6K, pS240/244 S6K
Tsika et al., 2014	R1441C TG mice (DAT prom)	22	**↔** LC3, p62	No	SNc	No	N/A
Liu et al., 2014	R1441G KI mice	18–22	**↔** LC3, Beclin-1	No		No	N/A
Yue et al., 2015	G2019S KI mice	15	↑ LC3II, **↔** p62	No	Whole brain	No	No gross morphological changes
Schapansky et al., 2018	G2019S KI mice	20	LAMP1, LC3I	No	CCx	No	N/A
Ho et al., 2018	G2019S TG mice (PDGF prom)	12-19	↑ p62, LC3	↑	Whole brain	N/A	N/A
Ho et al., 2019	R1441G KI mice	18	↑ LAMP2A, hsc70 (m.f.) ↑ GAPDH (m.f., c.f.)	↑	CCx, STR	No	N/A
Wallings et al., 2019	BAC G2019S rats	22	↑ LC3 puncta	N/A	SN	No	N/A
	BAC R1441C rats	22	↑ LC3 puncta		CCx SN		

*↑ increase, ↓ decrease, **↔** no change; AP, autophagosomes, c.f., cytosolic fraction; m.f., membrane fraction; NDG, nigrostriatal degeneration; prom, promoter; TG, transgenic.*

Autophagy abnormalities were observed also in R1441C/G LRRK2 mutants, although more inconsistently. A pattern similar to that observed in hG2019S KI mice was detected in 23–24 month-old hR1441C transgenic mice (Ramonet et al., 2011). Conversely, conditional transgenic mice expressing the R1441C mutation selectively in DA neurons, did not show accumulation of α-syn, tau or ubiquitin or even changes of p62 and LC3 levels at the age of 12–22 months (Tsika et al., 2014). This was confirmed also in R1441G KI mice, where no changes in autophagic markers LC3 and Beclin-1 were observed at 3 and 18–22 months, when compared with age-matched WT controls (Liu et al., 2014). Nonetheless, a more recent and comprehensive study in these mice at different ages spanning from 3 to 18 months, showed an age-dependent impairment of CMA (Ho et al., 2019). In particular, Western analysis revealed a significant increase in soluble α-syn and total amyloid-like α-syn oligomers in 18-month-old but not younger R1441G KI mice. Such increase of α-syn load was associated with lysosomal redistribution, with clustering of lysosomes around the nucleus of striatal neurons. Lysosomal impairment was confirmed by the (mild) increase of LAMP2A and Hsc70 levels in the membrane fraction of striatal lysates of 18-month-old mice, and by the accumulation of a typical CMA substrate, such as GAPDH. Further analysis in embryonic fibroblasts obtained from R1441G mice proved that lysosomal clearance of α-syn was indeed reduced. Interestingly, CMA activation rescued these changes, suggesting the therapeutic potential of this approach.

### Mechanisms Through Which LRRK2 Regulates Autophagy

**FIGURE 2:**
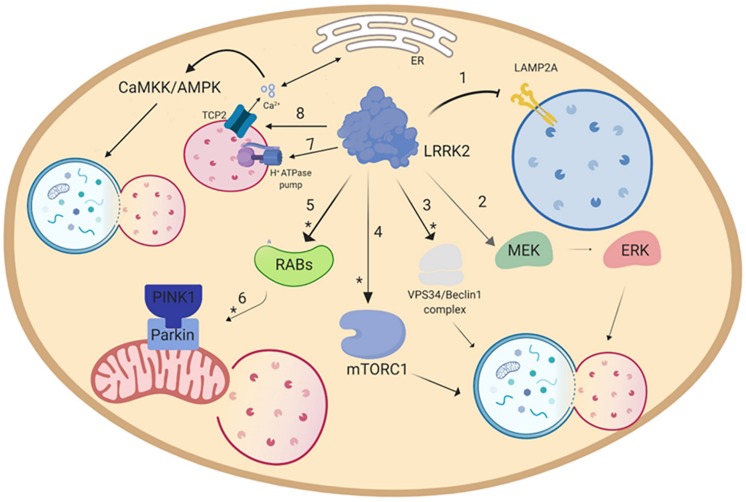
Putative pathways through which LRRK2 modulates autophagy. **(1)** Overexpression of LRRK2 or its mutants inhibits CMA through blockade of the translocation into the lysosome operated by the LAMP2A receptor (Orenstein et al., 2013). Furthermore, LRRK2 is able to modulate autophagy by activating either **(2)** the MEK/ERK (Bravo-San Pedro et al., 2013), or the **(3)** VPS34/Beclin1 complex (Manzoni et al., 2016). Other putative pathways are **(4)** the mTORC1 pathway (Imai et al., 2008; Ho et al., 2018), or **(8)** the Ca^2+^/CaMKK/AMPK pathway (Gomez-Suaga et al., 2012b). As for mitophagy **(5)**, LRRK2, via Rab activation (Wauters et al., 2019), is able to disrupt the **(6)** PINK1/Parkin-mediated mitophagy (Saez-Atienzar et al., 2014; Schapansky et al., 2014; Yakhine-Diop et al., 2014; Bonello et al., 2019). Furthermore, LRRK2 can regulate lysosomal pH via **(7)** lysosomal H^+^-ATPase pump (Wallings et al., 2019). Asterisk (^∗^) indicates the putative pathways modulated by pharmacological LRRK2 kinase inhibitors.

Over the years, different pathways have been identified through which LRRK2 modulates autophagy, although in most studies it is not possible to dissect out direct vs. indirect effects (Figure 2). LRRK2 might operate through various subsets of Rab GTPases, since Rab3A/B/C/D, Rab8A/B, Rab10, Rab12, Rab29, Rab35 and Rab43 are LRRK2 substrates (Ito et al., 2016; Steger et al., 2016, 2017) and the Rab GTPase network has been implicated in many stages of autophagy (Zoppino et al., 2010; Dou et al., 2013; Feldmann et al., 2017). Another putative pathway through which LRRK2 regulates autophagy is the MEK/ERK/Beclin-1 axis. In particular, Bravo-San Pedro et al. (2013) reported that LRRK2 G2019S increased basal autophagy in fibroblasts and the effect was reversed by the MAPK1/3 inhibitor UO126. Moreover, Manzoni et al. (2016) reported that activation of autophagy following LRRK2 kinase inhibition requires the increase of PI3P levels operated by the VPS34/Beclin-1 rather than mTOR/ULK1 pathway. Both studies are in line with the findings of Plowey et al. (2008) who showed that 3-Methyladenine, a PI3K inhibitor responsible for the mTOR-mediated activation of autophagy, did not prevent mutant LRRK2-induced neurite shortening, whereas the MAPK1/3 inhibitor U0126 did. In contrast with this view, activation of RAW264.7 macrophages or murine BV2 microglial cells via TLR4 caused phosphorylation and membrane translocation of LRRK2, inducing autophagy via an mTOR-dependent mechanism (Schapansky et al., 2014). The discrepancy on which pathway is preferentially recruited by LRRK2 for activating autophagy might be due to the different cell types employed. In fact, LRRK2 might exert a different effect on immune cells compared to other cell lines since immune cells express high levels of LRRK2 mRNA and protein, which are further enhanced in case of activation of these cells (Hakimi et al., 2011; Thevenet et al., 2011). Furthermore, cell activation and translocation of LRRK2 to the autophagosome membrane might be relevant for the physiological activity of LRRK2 but not for the pathological mechanisms underlying LRRK2-associated PD. Finally, work from Hilfiker and collaborators disclosed a link between calcium homeostasis, LRRK2 and autophagy (Gomez-Suaga and Hilfiker, 2012; Gomez-Suaga et al., 2012a, b). It is well known that cytosolic calcium regulates autophagy at different levels (Bootman et al., 2018). These authors reported that overexpression of LRRK2 or the G2019S mutant in cell lines increases the autophagosome number through an mTOR-insensitive, LRRK2 kinase-sensitive pathway (Gomez-Suaga et al., 2012b). The LRRK2 effect relied on Ca^2+^ release from ER and CaMKK/AMPK pathway activation, and was mimicked by the Ca^2+^ mobilizing compound nicotinic acid adenine nucleotide diphosphate (NAADP), acting via endolysosomal two-pore channels (TPCs). Interestingly, LRRK2 effect was occluded by an inactive mutant of TPC type 2 (TPC2) and by a pharmacological TPC2 inhibitor. Since TPC2 are expressed by lysosomes, it was proposed that LRRK2 causes the opening of TPC2 and Ca^2+^ release from lysosomes, which triggers further Ca^2+^ release from ER and induces autophagy via CaMKK/AMPK (Gomez-Suaga and Hilfiker, 2012). It remains to be established whether TPC2 opening is mediated by a direct interaction with LRRK2 or a recruitment of Rabs (e.g., Rab7).

LRRK2 seems also to play a significant role on more specific subtypes of autophagy, namely mitophagy and CMA. A link between LRRK2 and mitophagy was early suggested by two studies that showed the presence of TOM20, LC3 and active LRRK2 in the same iodixanol gradient membrane fraction of RAW264.7 macrophages and BV2 microglial cells (Schapansky et al., 2014). Moreover, pharmacological inhibition of LRRK2 kinase activity induced Drp-1 mediated mitochondrial fission in SH-SY5Y cells (Saez-Atienzar et al., 2014). More recently, Bonello et al. (2019) demonstrated that G2019S LRRK2 impairs PINK1/Parkin-mediated autophagy in human fibroblasts, and that LRRK2 kinase activity is instrumental to this effect. However, this process does not seem to be directly mediated by LRRK2 but rather by the interaction with Rab10. In normal conditions, Rab10 accumulates on depolarized mitochondria through the interaction with PINK1 and Parkin. Here, Rab10 binds to the autophagy receptor optineurin and promotes its accumulation and exposition, facilitating the degradation of depolarized mitochondria. LRRK2, in its physiological state and more prominently in the presence of kinase-enhancing mutations, phosphorylates Rab10 at the Thr73 residue, preventing its accumulation and thus inhibiting mitophagy (Wauters et al., 2019). Furthermore, such LRRK2-mediated inhibition of mitophagy makes the cell more susceptible to mitochondrial damage and subsequent cell death (Yakhine-Diop et al., 2014). A direct link between LRRK2 and lysosomal activity was recently pointed out by Wallings et al. (2019), showing that hWT LRRK2 interacts with the a1 subunit of the v-type H^+^ ATPase proton pump (vATPase a1). This pump regulates the acidity of the lysosomal lumen, which is crucial for the activity of lysosomal enzymes. Primary cortical neurons obtained from LRRK2 R1441C mice showed decreased binding and protein levels of vATPase a1, with a significant more basic lysosomal pH, leading to inhibition of the lysosomal degradation activity (Wallings et al., 2019).

Regarding CMA, similar to α-syn (Cuervo et al., 2004), LRRK2 bears different KFERQ peptide motifs in its amino acid sequence that can be targeted by hsc70. Furthermore, high levels of WT or LRRK2 mutants inhibit the formation of the CMA translocation complex at the lysosomal membrane, thus blocking CMA (Orenstein et al., 2013). The LRRK2-mediated blockage of CMA induces a compensatory increase in LAMP2A and accumulation of other CMA substrates, such as α-syn (Orenstein et al., 2013). The observation that also high levels or aberrant forms of α-syn inhibits CMA (Cuervo et al., 2004), strongly points toward a synergistic role of these two proteins in neurotoxicity and protein aggregation, which has been a hot topic in the last few years.

## Are ALP Changes Observed in LRRK2 Models Relevant for LRRK2-Induced Neurodegeneration?

*In vitro* studies have demonstrated that not just the G2019S but also the less common LRRK2 pathogenic mutants are associated with enhanced kinase activity (Zhao and Dzamko, 2019), and that LRRK2 kinase activity is instrumental to LRRK2-mediated toxicity (West et al., 2005; Greggio et al., 2006; Plowey et al., 2008; Yao et al., 2010). On this basis, LRRK2 kinase inhibitors are being investigated as disease-modifying agents in models of LRRK2-associated and idiopathic PD (West, 2017; Zhao and Dzamko, 2019). Therefore, investigating if and to which extent the neuroprotective effect of LRRK2 inhibitors is mediated through the modulation of ALP is of great relevance and translational potential.

It was originally suggested that autophagy dysregulation contributes to the LRRK2-associated neurotoxicity *in vitro*, since removal of key autophagic proteins LC3 or ATG7 prevented G2019S-associated neurite shortening in SH-SY5Y cells, whereas autophagy activation by rapamycin worsened it (Plowey et al., 2008). In other studies, autophagy or CMA activation was shown to be protective against LRRK2-associated toxicity. In fact, rapamycin rescued the increase of cell death triggered by an UPS inhibitor in G2019S-overexpressing HEK293T cells (Gomez-Suaga et al., 2012b). Likewise, CMA activation rescued the impairment of lysosomal activity, the reduction of LAMP2A levels and the accompanying increase of α-syn in mouse embryonic fibroblasts and primary neurons obtained from R1441G KI mice (Ho et al., 2019), although whether these effects made cells more resistant to parkinsonian toxins was not reported. Consistent with the view that autophagy blockade leads to neurodegeneration (Hara et al., 2006; Komatsu et al., 2006b), Wade-Martins and collaborators reported that the autophagy inhibitor bafilomycin A1 caused cell death under starvation conditions in HEK293T cells (Alegre-Abarrategui et al., 2009). In this process, LRRK2 plays a role since LRRK2 knock-down was protective (Alegre-Abarrategui et al., 2009).

Only a few studies specifically addressed whether LRRK2 kinase inhibitors modulate ALP *in vitro*, unfortunately leading to opposite conclusions. Manzoni et al. (2013a) demonstrated that LRRK2 inhibitors LRRK2-IN1, GSK2578215A and CZC-25146 increase the autophagic flux via an mTORC1-independent pathway, either acting via the Beclin-1 or directly impacting on ULK1 (Manzoni et al., 2016, 2018). Consistent with induction of autophagy, GSK2578215A elevated LC3II and reduced p62 levels in bone-marrow-derived macrophages, although the increase of LC3II levels was minimally affected by bafilomycin A1, leading the authors to hypothesize that such increase was not related to autophagic flux changes (Hartlova et al., 2018). These studies would suggest that blocking LRRK2 kinase activity improves the proteolytic events and helps cope with misfolded proteins and damaged organelles that can drive cell death. Consistently, Schapansky et al. (2018) reported that LRRK2-IN-1 and CZC25146 reversed the lysosomal changes and reduced the accumulation of detergent-insoluble α-syn observed in primary cortical neurons obtained from G2019S KI mice. Moreover, Bonello et al. (2019) showed that GSK2578215A and LRRK2-IN-1 were able to reverse the impairment of PINK1/parkin-mediated mitophagy induced by the G2019S LRRK2 mutation. In contrast with these studies, Galindo and collaborators showed that nanomolar (i.e., LRRK2 specific) concentrations of GSK2578215A impaired the autophagic flux by altering the autophagosome/lysosome fusion, which led to mitophagy, mitochondrial fission and cytotoxicity (Saez-Atienzar et al., 2014). A reduction of the autophagic flux following LRRK2 pharmacological blockade with LRRK2-IN-1 and GSK2578215A was also observed in microglial and monocytic cells, which was associated with an impairment in clearance of Q74 protein aggregates in microglial cells (Schapansky et al., 2014). The reasons why these two sets of studies reached opposite conclusions is unclear, and might depend on cell models and protocols adopted. In addition, first generation LRRK2 inhibitors were used in these studies. Major limitation was also that none of these studies was designed to investigate whether LRRK2 inhibitors were neuroprotective.

*In vivo* studies did not help solve the issue. In fact, studies proving that dysregulation of autophagy is instrumental to the LRRK2-associated synucleinopathy *in vivo* are lacking. This is in part due to the general difficulty in reproducing a parkinsonian phenotype in LRRK2 KO, KI or transgenic mice (Volta and Melrose, 2017). In fact, genetic deletion of LRRK2 is not associated with nigrostriatal DA neuron degeneration whereas overexpression of hG2019S or hR1441C/G LRRK2 is inconsistently associated with late-onset nigrostriatal degeneration (Volta and Melrose, 2017). G2019S KI mice also do not show overt nigrostriatal neurodegeneration (Volta and Melrose, 2017), although functional and morphological changes at dopaminergic synapses and alterations of DA release have been reported along with aging, which might represent early sign of neuronal demise or susceptibility factors to parkinsonism (Yue et al., 2015; Longo et al., 2017; Novello et al., 2018).

Consistently, when these mice are exposed to parkinsonian toxins, they do show enhanced susceptibility to nigrostriatal degeneration. Thus, Daher et al. (2015) reported that G2019S transgenic rats were more prone than wild-type rats to develop nigrostriatal dopaminergic degeneration after AAV-mediated α-syn overexpression, whereas Karuppagounder et al. (2016) showed that G2019S transgenic mice were more sensitive to acute MPTP toxicity. Interestingly, subchronic administration of the second-generation LRRK2 kinase inhibitor PF-06447475 (West, 2017) reversed the neurotoxicity of α-syn overexpression not only in G2019S transgenic but also in wild-type rats. Although, the authors did not investigate whether this effect was due to a reduced α-syn burden mediated by an increase of the proteolytic events, this study revealed that pharmacological LRRK2 kinase inhibition could represent an appealing therapeutic approach also to idiopathic PD. This was confirmed by Greenamyre and collaborators who reported that repeated rotenone administration caused pSer1292 LRRK2 phosphorylation (index of LRRK2 activation) and pSer129 α-syn accumulation (possible early index of neurotoxicity) in SNc neurons, along with loss of nigrostriatal DA neurons which were reversed by systemic administration of the third-generation (West, 2017) LRRK2 inhibitor PF-360 (Di Maio et al., 2018; Rocha et al., 2019). Conversely, Henderson et al. (2019) failed to observe neuroprotection and clearance of α-syn aggregates in naïve mice injected with α-syn preformed fibrils (PFF), and chronically administered with the potent third-generation LRRK2 kinase inhibitor MLi-2. This would suggest that the contribution of LRRK2 to nigrostriatal degeneration might depend on the neurotoxicity pathways recruited in a specific experimental model of PD. Despite the role of LRRK2 inhibitor in idiopathic PD remains to be firmly established, these models offer the opportunity to elucidate whether ALP modulation contributes to the neuroprotection provided by LRRK2 inhibitors.

It is important to notice that these studies were performed in young-adult (i.e., 3-month-old) mice. Aged animals, however, might represent a more appropriate model to investigate the contribution of ALP to LRRK2-induced neurodegeneration. Indeed, aging is associated with autophagy and CMA impairment, and *in vivo* studies suggest that aging facilitates LRRK2-induced neurodegeneration. In fact, some studies in hG2019S and hR1441C transgenic mice reported a 18–50% loss of nigral DA neurons at old ages (16–21 months) (Ramonet et al., 2011; Chen et al., 2012; Weng et al., 2016) which were associated with morphological changes of AV and autophagosomes, reminiscent of ALP impairment (Ramonet et al., 2011). The view that aging might facilitate the onset of G2019S LRRK2 neurotoxicity might be further corroborated by the finding that virus-mediated overexpression of hG2019S LRRK2 in the striatum of C57BL/6J mice caused marked neuroinflammation associated with striatal cell loss (but not dopaminergic terminal loss) in 19-month-old but not 9–10-week-old mice (Kritzinger et al., 2018). Although quantification of these changes was not provided, qualitative analysis of microphotographs revealed that p62 accumulation was greater in aged mice, suggesting that impairment of autophagy might play a role in the underlying neurotoxic mechanisms. Changes in ALP markers (increase in LC3 puncta) were also observed in old BAC G2019S and BAC R1441C mice in the absence of overt neurodegeneration, suggesting that changes in ALP might precede neurodegeneration (Wallings et al., 2019).

Studies in G2019S KI mice carried out by Morari and collaborators pointed out an increase of α-syn pSer129 levels in the striatum and SNpc at 12 months but not earlier ages (Longo et al., 2017; Novello et al., 2018). Despite the α-syn signal is due to soluble forms of α-syn (Novello et al., 2018), it might reflect changes in proteostasis, and particularly a reduction of autophagic flux due to G2019S mutation (Schapansky et al., 2018). Consistently, when injected with an AAV2/9 overexpressing human A53T α-syn under the synapsin I promoter (Bourdenx et al., 2015; Arcuri et al., 2016), the aged animals showed a greater reduction of nigral DA neurons associated with greater accumulation of insoluble, PK-resistant α-syn and pSer129 α-syn aggregates in SNpc (Novello et al., 2018). This might reflect a reduced ability of old G2019S KI animals to cope with α-syn overload, as shown in old nematodes (Saha et al., 2015). LRRK2 mutations and aging will therefore work synergistically to inhibit ALP and, consequently, α-syn clearance, thereby facilitating disease progression and spreading; in this respect impaired autophagy has been viewed as an “aggravator” of PD (Johnson et al., 2019). These *in vivo* studies would therefore provide a rationale for pharmacological inhibition of LRRK2 kinase activity both in relation to neuroprotection and autophagy modulation.

We should also mention the careful *in vivo* work done by Giaime et al. (2017), showing that, different from LRRK2 or LRRK1 KO mice, DKO mice develop age-dependent DA neuron loss and ALP changes, which became significant at 14–15 months of age. Lack of both LRRK proteins also affected the viability of noradrenergic cells in locus coeruleus noradrenergic and striatal GABAergic medium-sized spiny neurons, and was associated with elevation of α-syn levels in striatum. Since changes in ALP markers preceded neuronal loss, these authors proposed that impairment of autophagy was instrumental to neurodegeneration (Giaime et al., 2017).

Finally, the role of impaired autophagy/CMA in PD may go beyond α-syn clearance, and changes in ALP might play a wider role in PD than hitherto imagined. Indeed, autophagy is active not only at the soma but also at the synaptic terminals, where it regulates synaptic protein quality control and synaptic activity (Hernandez et al., 2012; Vijayan and Verstreken, 2017). Soukup et al. (2016) have shown that protein quality control at synapses of *Drosophila melanogaster* is mediated by EndophilinA, a protein enriched in phagophore membranes, which is phosphorylated by LRRK2. LRRK2 phosphorylation allows the protein to create curved membranes that can harbor and recruit ATG3 to initiate autophagy (Soukup et al., 2016). Therefore, derangement of synaptic autophagy can affect synaptic homeostasis and plasticity which may increase the susceptibility to PD in the long term (Soukup et al., 2016, 2018; Vijayan and Verstreken, 2017).

## Conclusion and Perspectives

Autophagy is a key mechanism through which cells operate protein quality control and respond to environmental challenges. Autophagy activation can prolong the life-span of invertebrates and rodents, whereas impairment of autophagy leads to a number of disorders, among which neurodegenerative disorders of aging. A close association between autophagy and neurodegeneration has been established, although the casual link remains to be proven. In PD, ALP impairment can lead to increased levels of misfolded α-syn, accelerating its transport and spread throughout the brain. Aging, the major risk factor in PD, might facilitate this process since the efficiency of ALP machinery worsens over time. Genetic risk factors of PD, such as LRRK2 mutations, also impact on autophagy, although the control operated by LRRK2 and its mutants on ALP machinery is far from clear. *In vitro* studies offer evidence that G2019S LRRK2 causes autophagy and CMA impairment, and that autophagy activation rescues LRRK2-associated cell death or toxicity. Unfortunately, ALP changes measured in LRRK2 KO, KD and LRRK2 mutant transgenic mice are inconsistent, perhaps due the intrinsic complexity of the network through which LRRK2 controls autophagy, and the lack of standardized protocols to study autophagic flux *in vivo*. Furthermore, data obtained in transgenic mice should be taken with caution due to the obvious limitations of the models (e.g., non physiological expression levels of LRRK2, coexistence of endogenous and mutant LRRK2, neuronal pattern of LRRK2 expression). Finally, the lack of solid, progressive *in vivo* models of LRRK2-associated synucleinopathy has hindered the comprehension of the role of autophagy in LRRK2-associated parkinsonism, and discouraged the testing of potential disease-modifying agents, among which LRRK2 kinase inhibitors. The identification of early markers of neuronal demise in LRRK2 mice, among which alterations in presynaptic autophagy, will offer new opportunities for pharmacological intervention.

## Author Contributions

All authors listed have made a substantial, direct and intellectual contribution to the work, and approved it for publication.

## Conflict of Interest

The authors declare that the research was conducted in the absence of any commercial or financial relationships that could be construed as a potential conflict of interest.

## Abbreviations

4E-BP: eukaryotic initiation factor 4E binding protein
α-syn: α-synuclein
AD: Alzheimer’s disease
ALP: autophagy-lysosomes pathway
AMPK: 5′-AMP-activated kinase
ATG: autophagy-related gene 8
AV: autophagic vacuoles
A β: β amyloid
CMA: chaperone-mediated autophagy
CNS: central nervous system
COR: C-terminal of Roc
DA: dopamine
DAMPs: damage-associated molecular patterns
DKO: double knock-out
DLB: dementia with Lewy bodies
eIF4E: eukaryotic initiation factor 4E
EM: electron microscopy
ER: endoplasmic reticulum
ESCRT: endosomal sorting complexes required for transport
GCase: β-glucocerebrosidase
HD: Huntington’s disease
Hsc70/HSPA8: heat shock 70 kDa protein 8
IGF-1: insulin growth factor-1
KD: kinase dead
KI: knock-in
KO: knock-out
LAMP1: lysosome-associated membrane protein 1
LAMP2A: lysosome-associated membrane protein 2 isoform A
LBs: Lewy bodies
LC3I: microtubule-associated protein 1 light chain 3 beta isoform 1
LC3II: microtubule-associated protein 1 light chain 3 beta isoform 2
LIR: LC3-interacting region
LRRK1: leucine-rich repeat kinase 1
LRRK2: leucine-rich repeat kinase 2
mtDNA: mitochondrial DNA
mTORC1: mammalian target of rapamycin complex 1
MVBs: multivesicular bodies
NFE2L2/NRF2: nuclear factor, erythroid derived 2, like 2
PD: Parkinson’s Disease
PINK1: PTEN-induced putative kinase 1
ROC: Ras of complex
ROS: reactive oxygen species
SNpc: substantia nigra pars compacta
SQSTM1/p62: Sequestosome 1
TDP43: TAR DNA-binding protein 43
TFEB: transcription factor EB
TRIM: tripartite motif protein family
UBA: ubiquitin-associated domain
ULK1: uncoordinated-51-like kinase 1
UPS: ubiquitin-proteasome system
VPS34: vacuolar protein sorting 34.
